# The kynurenine pathway in RNA virus infection: immunometabolic control of antiviral defense and viral persistence

**DOI:** 10.3389/fimmu.2026.1923351

**Published:** 2026-09-07

**Authors:** Daniela Nahomi Calderón-Sandate, Manuel Josafat Huerta-Garza, Blanca Azucena Márquez-Reyna, David Mauricio Cañedo-Figueroa, Ximena Hernández-Rodríguez, Flor Itzel Lira-Hernández, Juan Fidel Osuna-Ramos, Jaime Cardoso-Ortiz, Rosa María del Ángel, Alan Orlando Santos-Mena, Luis Adrián De Jesús-González

**Affiliations:** 1 Laboratorio de Virología Molecular, Unidad de Investigación Biomédica de Zacatecas, Instituto Mexicano del Seguro Social, Zacatecas, Mexico; 2 Department of Infectomics and Molecular Pathogenesis, Center for Research and Advanced Studies of the National Polytechnic Institute, Mexico City, Mexico; 3 Laboratorio de Virología y Diseño de Antivirales, Facultad de Medicina, Universidad Autónoma de Sinaloa (UAS), Culiacan, Mexico; 4 Unidad Académica de Ciencias Químicas, Universidad Autónoma de Zacatecas, Zacatecas, Mexico

**Keywords:** antiviral immunity, aryl hydrocarbon receptor, host-directed antiviral therapy, IDO1, immunometabolism, RNA viruses, tissue repair, tryptophan-kynurenine pathway

## Abstract

RNA virus infections are shaped by the interplay among viral replication, innate immune sensing, and host metabolism, which together determine the magnitude, duration, and quality of antiviral responses. The tryptophan–kynurenine (Trp–Kyn) pathway has emerged as an important immunometabolic axis linking interferon-driven inflammation, amino acid availability, immune-cell function, tissue homeostasis, and viral persistence. This review examines the roles of indoleamine 2,3-dioxygenase 1 (IDO1), IDO2, tryptophan 2,3-dioxygenase (TDO), Trp depletion, general control nonderepressible 2 (GCN2) signaling, mechanistic target of rapamycin complex 1 (mTORC1), Kyn-derived metabolites, and aryl hydrocarbon receptor (AhR) activation in host responses to RNA virus infection. We integrate genetic, pharmacological, cellular, animal, and clinical evidence across positive-sense and negative-sense single-stranded RNA viruses, double-stranded RNA viruses, and reverse-transcribing RNA viruses, including SARS-CoV-2, dengue virus, Zika virus, hepatitis C virus, influenza A virus, respiratory syncytial virus, rotavirus, reovirus, HIV-1, and SIV. Across these systems, the Trp–Kyn–AhR axis can influence interferon responses, viral replication, immune-cell function, tissue injury, and disease outcome. EMCV myocarditis provides causal proof of principle, whereas other RNA-virus models provide complementary functional and clinical evidence. Importantly, pathway activation does not confer a uniform antiviral or pathogenic phenotype; its biological consequences depend on viral class, tissue tropism, viral burden, inflammatory intensity, cell type, and disease stage. On this basis, we propose a time-phase model in which early or excessive IDO1–Kyn–AhR signaling may impair type I interferon responses, NK-cell function, CD8+ T-cell expansion, and viral clearance, whereas appropriately regulated activation during later inflammatory or resolution phases may limit immunopathology, support epithelial repair, and preserve tissue homeostasis. Persistent or dysregulated activation may instead contribute to exhaustion-like immune states, chronic inflammation, neuroimmune dysfunction, and post-acute viral sequelae. Finally, we discuss phase-adapted therapeutic strategies, including IDO1 inhibition, AhR agonism or antagonism, KMO-directed modulation, Trp-based entry inhibitors, and biomarker-guided combination approaches. Together, these findings position the Trp–Kyn–AhR axis as a dynamic, context-dependent target for precision host-directed antiviral immunometabolism.

## 1 Introduction

RNA viruses are among the most active categories of human diseases, characterized by their rapid replication, substantial genetic diversity, and robust ability to adapt to immunological and therapeutic challenges. A primary factor influencing this plasticity is the restricted proofreading ability of numerous viral RNA-dependent polymerases, which promotes the ongoing production of mutant spectra during replication. The combination of rapid replication cycles and substantial viral population sizes allows RNA viruses to navigate adaptive landscapes, promoting immune evasion, antiviral resistance, host switching, and prolonged persistence (1, 2). These characteristics are fundamental to the biology of clinically significant RNA viruses, such as influenza A virus (IAV), dengue virus (DENV), Zika virus (ZIKV), respiratory syncytial virus (RSV), coronaviruses, hepatitis C virus (HCV), and human immunodeficiency virus type 1 (HIV-1), all of which exemplify how viral evolution and host immune pressure influence infection outcomes (3–5).

While RNA virus infections have historically been examined in relation to innate and adaptive immunity, it is now clear that the metabolic condition of both infected cells and immune populations significantly impacts antiviral defense. Immunometabolism has emerged as a conceptual framework that elucidates how metabolic pathways regulate immune cell activation, differentiation, effector functions, and exhaustion. Metabolic changes actively influence the extent and quality of antiviral responses, rather than only serving as passive consequences of inflammation (6, 7). During infection, immune cells undergo significant metabolic reprogramming to support cytokine synthesis, clonal proliferation, antigen presentation, and cytotoxic function. Activated lymphocytes and inflammatory macrophages frequently enhance aerobic glycolysis to rapidly produce ATP and biosynthetic precursors, whereas resting or regulatory cells predominantly rely on oxidative phosphorylation and fatty acid metabolism (8–10).

These alterations may promote viral replication while concurrently enhancing inflammatory pathways via mechanisms such as reactive oxygen species generation and nuclear factor kappa B (NFκB) activation (11, 12). The metabolic response to infection can either facilitate antiviral immunity or foster an environment conducive to viral persistence and immunological dysfunction, contingent upon timing, tissue context, and the severity of inflammation (13, 14). In this context, amino acid metabolism has become particularly significant within this framework, as amino acids function not only as substrates for protein synthesis but also as immunological signals that modulate cell proliferation, stress responses, and inflammatory tone (6, 15).

Tryptophan (Trp) exemplifies this principle prominently. Trp, an essential amino acid, is required for protein synthesis and serves as a precursor for several bioactive pathways involved in immunological control, brain function, redox balance, and tissue homeostasis. Trp metabolism is primarily distributed among three interconnected pathways: the serotonin pathway, which facilitates the production of serotonin and melatonin; the indole pathway, which produces various AhR ligands derived from microbiota and the host; and the kynurenine (Kyn) pathway, which is responsible for the majority of systemic Trp degradation and yields metabolites with immunoregulatory and metabolic functions, including Kyn, kynurenic acid, quinolinic acid, and nicotinamide adenine dinucleotide precursors (16–18).

The Kyn pathway has gained significance in viral immunology due to its high induction by inflammatory and interferon-mediated signals. The route commences with Trp-catabolizing enzymes, namely indoleamine 2,3-dioxygenase 1 (IDO1), indoleamine 2,3-dioxygenase 2 (IDO2), and tryptophan 2,3-dioxygenase (TDO2). IDO1 is the most inducible enzyme in inflammatory tissues. It is frequently elevated in response to interferon-gamma (IFN-γ), type I interferons, Toll-like receptor signaling, interleukin-1, and various inflammatory mediators produced during infection (19, 20). Upon activation, IDO1 diverts Trp metabolism towards Kyn synthesis, thereby reducing local Trp availability and elevating levels of metabolites that can alter immunological responses (15, 21).

Encephalomyocarditis virus (EMCV) myocarditis provides strong proof of principle for the role of the Kyn pathway in RNA virus infection. In Ido1−/− mice and in wild-type mice treated with 1-methyl-D, L-tryptophan, disruption of IDO1 activity enhanced type I interferon responses, reduced the cardiac viral burden, and improved survival. Administration of Kyn-pathway metabolites reversed this protection. Bone marrow chimera experiments supported a contribution from hematopoietic cells, and Kmo deficiency independently reduced mortality. Together, these findings demonstrate that direct perturbation of the pathway can modify both RNA virus control and disease outcome (22, 23).

Beyond EMCV, complementary evidence has emerged from genetic and pharmacological studies in IAV and LP-BM5; functional perturbation of IDO1, KMO, or AhR in ZIKV, coronaviruses, and CSFV; cell-based studies in RSV and HCV; and clinical metabolomic or immunological associations in COVID-19, DENV, influenza, and HIV/SIV. Although these models do not produce an identical phenotype, collectively they indicate that distinct nodes of the Trp-Kyn-AhR axis can influence viral replication, interferon responses, immune-cell function, and tissue injury (19, 24–28).

A central unresolved question is therefore not simply whether the pathway participates in antiviral immunity, but when and under which biological conditions its activity is protective or detrimental. Depending on the infection stage and tissue context, pathway activation may constrain antiviral immune pressure, limit immunopathology, or contribute to tissue adaptation and repair. Distinguishing among these functions is particularly important for defining the timing and tissue specificity of therapeutic interventions targeting IDO1, KMO, or AhR (29–33).

**Figure 1:**
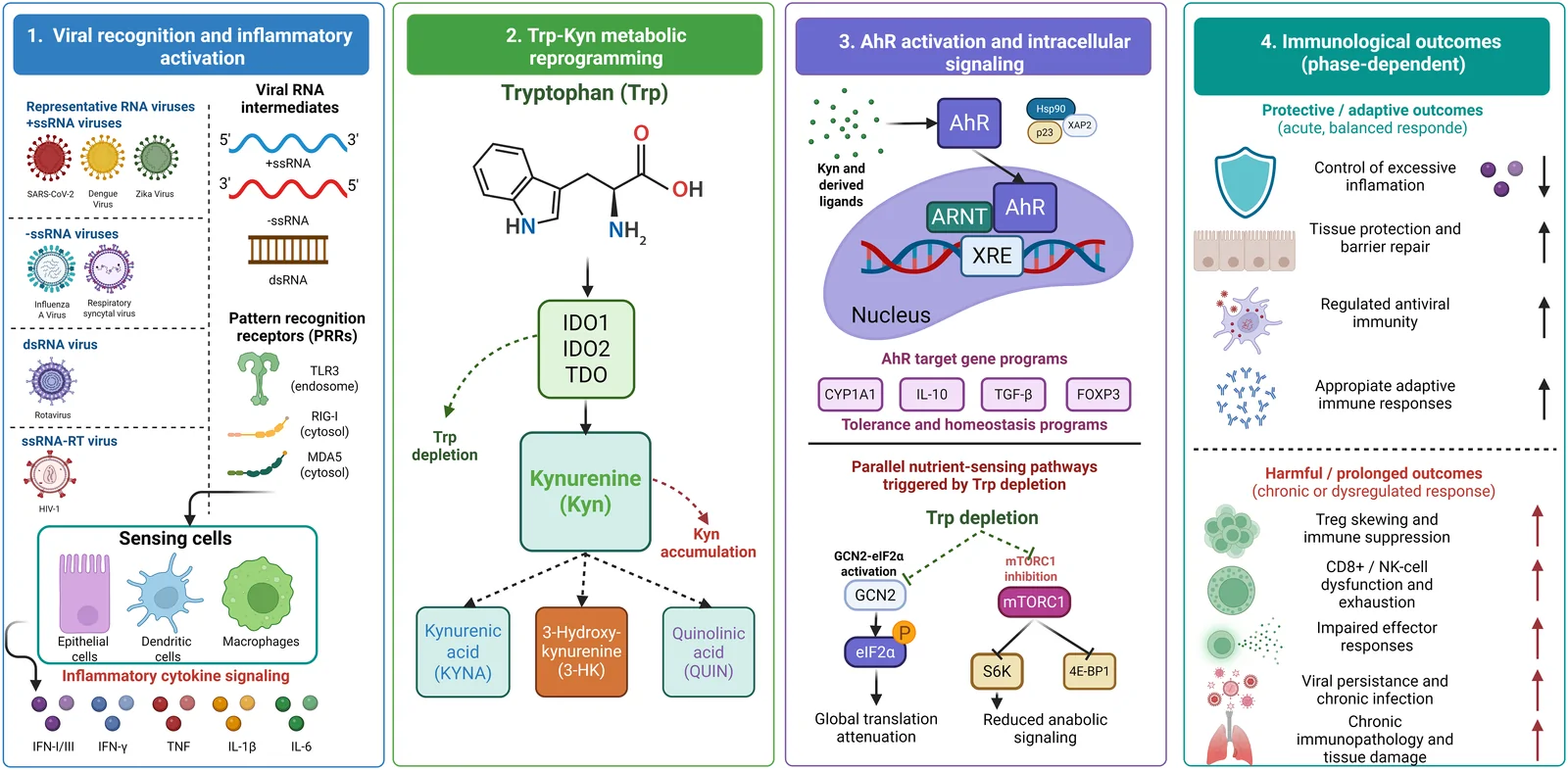
Overview of the Trp–Kyn–AhR immunometabolic axis during RNA virus infection. **(1)** Viral RNA recognition by pattern-recognition receptors (PRRs), including RIG-I, MDA5, and TLRs, triggers inflammatory cytokines and interferons that activate immunometabolic responses in epithelial cells, dendritic cells, macrophages, and other immune cells. **(2)** Tryptophan (Trp) is catabolized by the rate-limiting enzymes IDO1, IDO2, and TDO, yielding kynurenine (Kyn) and downstream metabolites such as kynurenic acid (KYNA), 3-hydroxykynurenine (3-HK), and quinolinic acid (QUIN). Trp depletion and Kyn accumulation constitute the central metabolic switch of the pathway. **(3)** Kyn and related metabolites activate the aryl hydrocarbon receptor (AhR), promoting AhR–ARNT nuclear translocation and the transcription of immune-regulatory genes. Simultaneously, Trp depletion activates GCN2 and suppresses mTORC1 signaling, thereby coordinating nutrient sensing, translational control, and cellular stress adaptation. **(4)** Integration of these signaling pathways regulates immune-cell differentiation and function. Balanced activation promotes controlled antiviral immunity and tissue homeostasis, whereas sustained activation promotes regulatory immune programs, impairs effector-cell activity, promotes immune tolerance, and favors viral persistence.

Accordingly, this review examines the Kyn pathway as a dynamic and temporally regulated immunometabolic axis during RNA virus infection. We first outline the molecular mechanisms through which interferon signaling, Trp depletion, GCN2 activation, and Kyn-mediated AhR signaling shape antiviral immunity. We then critically examine evidence across major RNA virus infections, including SARS-CoV-2, IAV, DENV, ZIKV, RSV, HCV, and HIV, identifying not only the principal mechanistic and translational findings but also the major gaps in knowledge that remain across these infections. These unresolved questions are discussed as opportunities for future research, particularly for defining when and how modulation of the Trp-Kyn-AhR axis could be exploited for antiviral development or as a host-directed therapeutic strategy. Finally, we propose a therapeutic-window model in which the biological consequences of targeting this axis depend on infection timing, viral burden, inflammatory severity, and tissue-specific immune regulation. The overarching immunometabolic framework, current knowledge gaps, and therapeutic opportunities discussed throughout this review are summarized in **Figure 1**.

## 2 Immunometabolic regulation of the tryptophan-kynurenine pathway

Viewing the Kyn pathway as a time-dependent immunometabolic switch raises a central mechanistic question: how does Trp metabolism translate inflammatory sensing into altered antiviral immunity? During RNA virus infection, pattern-recognition receptors, interferons, inflammatory cytokines, nutrient-sensing pathways, and tissue-repair programs act in concert to shape host responses (6, 15). Within this network, the Trp-Kyn pathway links inflammatory signaling to metabolic and transcriptional regulation of innate and adaptive immunity (21) through three interconnected processes: cytokine-driven IDO1 induction, nutrient-stress signaling triggered by Trp depletion, and receptor-mediated immune regulation by Kyn metabolites. The resulting effects vary according to cell type, tissue, infection stage, and the virological or pathological endpoint examined (34–36).

### 2.1 Interferon-driven activation of IDO1

IDO1 catalyzes the first and rate-limiting step of extrahepatic Trp degradation through the Kyn pathway (37, 38), linking antiviral cytokine signaling to metabolic restriction and immune-cell reprogramming (15, 21). Among its inflammatory inducers, IFN-γ is the most potent physiological regulator. Produced mainly by activated T lymphocytes and NK cells, IFN-γ signals through IFNGR1/IFNGR2 to activate the JAK1/JAK2-STAT1 pathway. Phosphorylated STAT1 translocates to the nucleus and, together with IRF1, promotes transcriptional programs involved in antimicrobial defense, antigen presentation, immune regulation, and IDO1 expression (39, 40).

The IFN-γ-JAK-STAT1-IRF1 axis induces IDO1 particularly in macrophages and dendritic cells, although epithelial, endothelial, microglial, and other tissue-resident cells can also express IDO1 under inflammatory conditions, enabling local regulation at sites of viral replication and tissue injury (38, 40). Once induced, IDO1 simultaneously reduces local Trp availability and increases Kyn and other bioactive metabolites, thereby altering the metabolic and signaling environment of immune cells (37, 38). These effects are especially relevant to activated lymphocytes, which require adequate amino-acid availability for clonal expansion, cytokine production, and cytotoxic function (21, 41).

The consequences of IDO1 activation during viral infection are context- and time-dependent. Early activity may limit excessive inflammation and tissue damage, whereas sustained activation can promote an immunoregulatory environment associated with impaired CD8+ T-cell and NK-cell function, regulatory T-cell expansion, and ineffective persistent inflammation (42, 43). This duality is particularly evident in SARS-CoV-2 infection, in which enhanced Trp catabolism and Kyn-pathway activity have been associated with systemic inflammation and disease severity (44, 45).

Beyond Trp catabolism, IDO1 can also exert non-catalytic signaling functions that reinforce tolerogenic programs (46). This distinction is therapeutically relevant because inhibiting IDO1 enzymatic activity may not fully reverse the immunoregulatory effects associated with IDO1 expression (32, 46).

### 2.2 Tryptophan depletion and GCN2-mediated translational control

Trp depletion acts as a nutrient-stress signal, particularly in activated lymphocytes, whose clonal expansion and effector differentiation depend on sustained amino-acid availability and protein synthesis (36, 41). Amino-acid limitation leads to accumulation of uncharged tRNAs, activating GCN2, which phosphorylates eIF2α to suppress global translation while initiating the integrated stress response. Persistent GCN2 signaling can consequently impair T-cell proliferation and cytokine production, directly linking inflammatory Trp catabolism to altered antiviral effector function (36, 47).

Despite global translational repression, selective ATF4 translation maintains adaptive programs involved in amino-acid transport, redox homeostasis, and cell survival, with effects that differ between antigen-presenting cells and expanding virus-specific lymphocytes. In parallel, Trp depletion attenuates mTORC1-dependent anabolic and effector programs, allowing GCN2 and mTORC1 to integrate the same metabolic perturbation into coordinated stress adaptation and reduced cellular growth (48, 49).

Thus, this nutrient-sensing arm should be interpreted according to its cellular and virological consequences rather than as intrinsically antiviral or immunosuppressive. Concurrently, Kyn accumulation engages the receptor-mediated regulatory arm described below (32, 38, 41).

### 2.3 Kynurenine-mediated AhR activation and immune reprogramming

Kyn and other Trp-derived metabolites activate AhR, which translocates to the nucleus, heterodimerizes with ARNT, and regulates xenobiotic-response-element-containing genes. AhR signaling can reshape both innate and adaptive immunity by promoting regulatory CD4+ T-cell programs and modulating antigen presentation, cytokine production, macrophage polarization, and dendritic-cell function. Depending on context, these effects may limit inflammatory damage but also weaken antiviral immune pressure (35, 50).

Sustained AhR activation may additionally impair cytotoxic programs, although mechanisms inferred from cancer-associated exhaustion require direct validation in RNA virus infection (31, 51). Functional studies in ZIKV and coronaviruses further indicate that AhR can alter viral replication and interferon responses in a model-dependent manner, emphasizing the need for direct perturbation studies rather than mechanistic extrapolation alone (27, 28). Importantly, AhR signaling is not inherently detrimental. In endothelial, epithelial, and mucosal compartments, it can support barrier integrity, tissue repair, and control of inflammatory injury. These protective effects should therefore be evaluated together with virological outcomes when defining the role of AhR during infection (52).

### 2.4 The dual role of the IDO1-kynurenine-AhR axis

**Figure 2:**
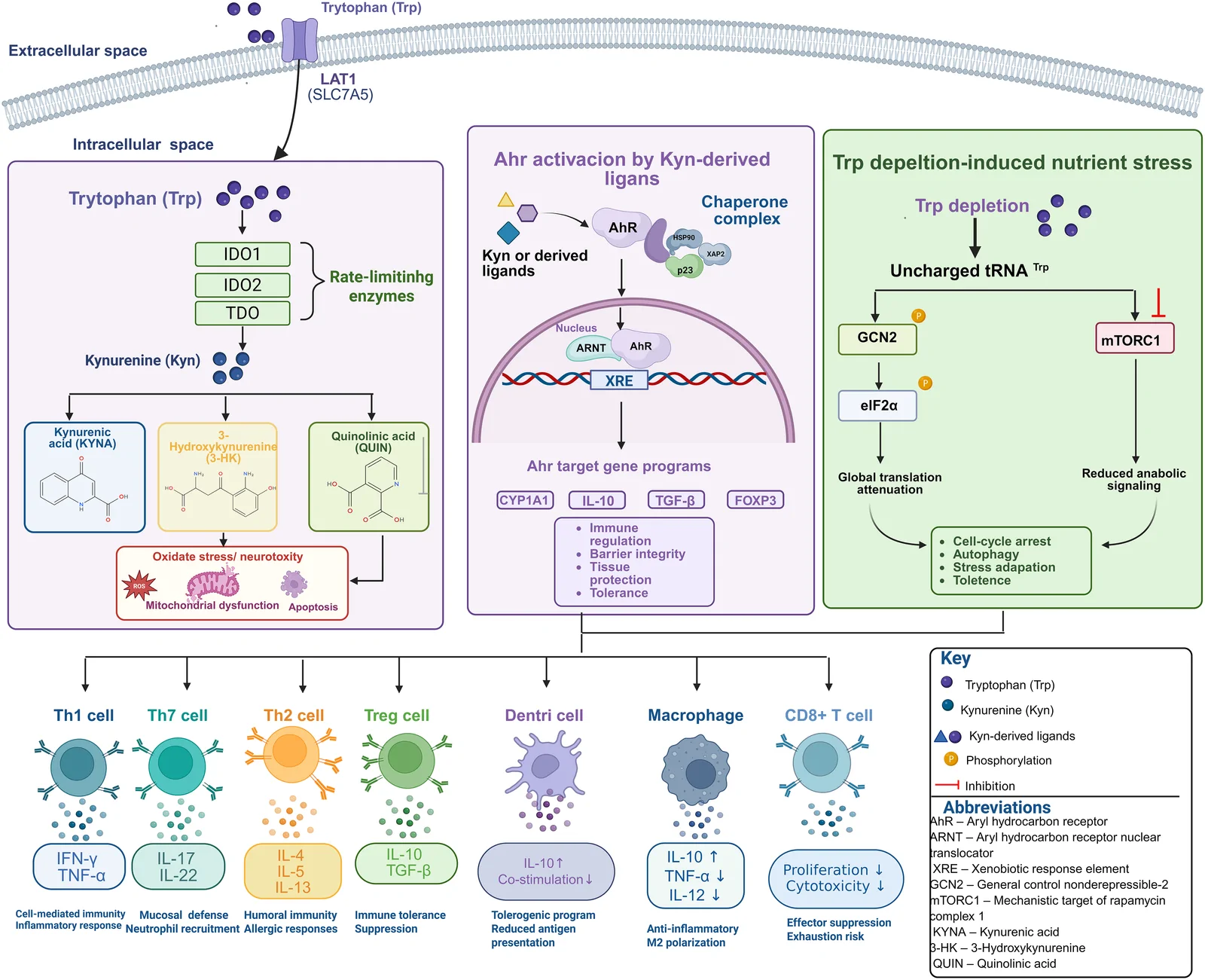
Mechanistic integration of Trp–Kyn–AhR signaling and nutrient sensing during RNA virus infection. Tryptophan (Trp) availability and catabolism coordinate complementary metabolic and transcriptional programs that regulate antiviral immunity. Trp is transported into cells and metabolized via the kynurenine pathway by IDO1, IDO2, and TDO, yielding kynurenine (Kyn) and downstream metabolites, including kynurenic acid (KYNA), 3-hydroxykynurenine (3-HK), and quinolinic acid (QUIN). Kyn-derived ligands activate the aryl hydrocarbon receptor (AhR), driving AhR–ARNT nuclear translocation and transcriptional programs that regulate immune regulation, barrier integrity, tissue protection, and tolerance. In parallel, Trp depletion triggers GCN2–eIF2α-dependent nutrient-stress signaling and dampens mTORC1-dependent anabolic programs. Integration of these pathways modulates the differentiation and function of T-cell subsets, dendritic cells, macrophages, and CD8+ T cells, linking inflammatory Trp catabolism to context-dependent antiviral and immunoregulatory outcomes.

The IDO1-Kyn-AhR axis exerts context- and phase-dependent effects during RNA virus infection. Excessive or sustained activation may impair antiviral effector responses, whereas appropriately localized signaling may limit immunopathology and preserve tissue function (23, 29, 30). During the viral-control phase, studies should determine whether modulation of IDO1, GCN2/mTORC1, or Kyn-AhR signaling alters interferon responses, NK-cell activity, antigen presentation, CD8+ T-cell expansion, or viral burden (27, 28). During inflammatory and resolution phases, these same nodes should be evaluated for their effects on cytokine-mediated injury, barrier restoration, fibrosis, and tissue repair (15, 42). This temporal framework provides a testable model rather than assuming a uniform pattern across RNA virus infections. The interconnected molecular mechanisms linking Trp catabolism, AhR signaling, nutrient sensing, and immune-cell function are summarized in **Figure 2**.

## 3 The kynurenine pathway across RNA virus infections

**Figure 3:**
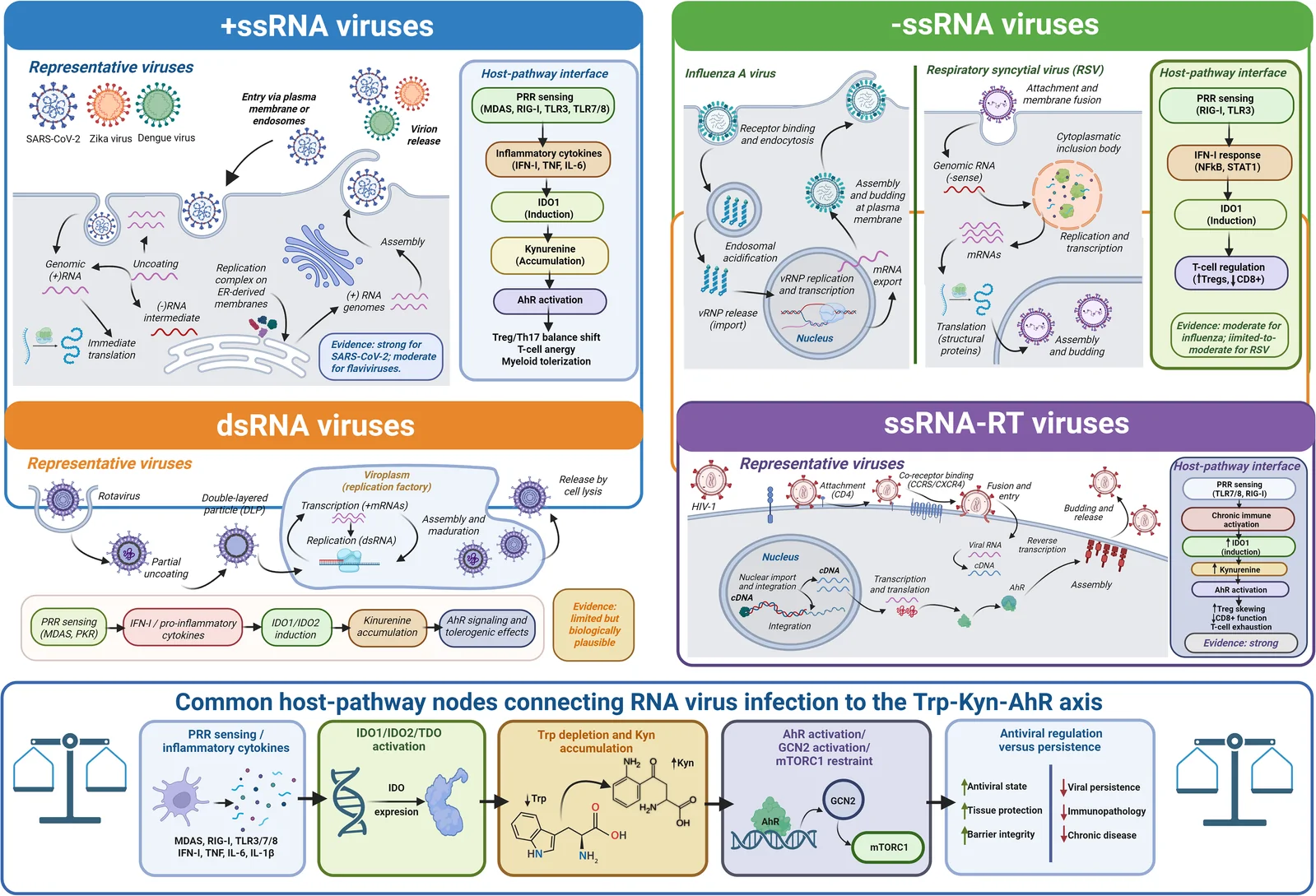
RNA virus classes and their interface with the Trp–Kyn–AhR axis. Comparative overview of the major RNA virus classes discussed in this review and their interactions with the Trp–Kyn–AhR immunometabolic pathway. Positive-sense single-stranded RNA viruses (+ssRNA), negative-sense single-stranded RNA viruses (−ssRNA), double-stranded RNA viruses (dsRNA), and reverse-transcribing RNA viruses (ssRNA-RT) share host signaling pathways despite major differences in genome organization and replication strategies. Viral sensing induces inflammatory cytokines that stimulate IDO1 expression, promoting Trp depletion, Kyn accumulation, AhR activation, and downstream immunometabolic reprogramming. The strength of experimental evidence supporting these mechanisms varies across viral families and is summarized for each viral class.

The molecular framework above supports a central conclusion: the Kyn pathway is a biologically relevant component of antiviral immunity whose contribution is best established by perturbation studies and then extended through cellular and clinical evidence. In this review, evidence is considered strongest when genetic deletion, pharmacological modulation, or metabolic rescue changes viral replication, interferon responses, survival, immune function, or tissue injury; functional *in vitro* studies and longitudinal clinical associations provide complementary levels of support. The Baltimore framework is retained to preserve virological comparability, while models within each class are discussed from the strongest available evidence toward emerging knowledge gaps. The interactions between the different RNA virus classes and the Trp–Kyn–AhR pathway are summarized in **Figure 3**.

### 3.1 Positive-sense single-stranded RNA viruses: causal, functional, and clinical evidence

EMCV, a picornavirus, provides the strongest causal example in the +ssRNA class. Ido1−/− mice and wild-type mice treated with 1-methyl-D,L-tryptophan showed increased survival, reduced cardiac viral burden, and increased type I interferon production. Administration of Kyn-pathway metabolites reversed this protection, and bone-marrow chimera experiments supported a hematopoietic contribution. This genetic–pharmacological–rescue sequence demonstrates that IDO1-derived metabolites can directly determine antiviral interferon activity and disease outcome in an RNA-virus model. EMCV also provides evidence at a downstream enzymatic node: Kmo−/− mice experienced lower mortality after infection. Together, the IDO1 and KMO studies establish EMCV as a proof-of-principle system for dissecting how individual Kyn-pathway branches influence viral control, inflammation, and survival (22, 23).

SARS-CoV-2 is a clear clinical model linking Trp metabolism to systemic inflammation. Multiple metabolomic studies and meta-analytic evidence indicate that COVID-19 is associated with reduced Trp, increased Kyn, and elevated Kyn/Trp ratios, particularly in severe disease. Because the Kyn/Trp ratio is commonly used as an indirect indicator of IDO1 activity, these findings support the interpretation that SARS-CoV-2 induces a strong immunometabolic response linked to inflammatory burden and immune dysfunction rather than viral replication alone (25, 53).

COVID-19 provides convergent clinical and mechanistic evidence for Kyn-pathway activation, whose biological meaning depends on whether it reflects counter-regulation or sustained immune suppression. On the other hand, sustained IDO1 activity may reduce Trp availability for proliferating lymphocytes, trigger general GCN2-dependent stress responses, and favor regulatory or exhaustion-like immune states that weaken antiviral pressure. This dual interpretation aligns with the broader immunometabolic role of IDO1 as both an antimicrobial/metabolic stress pathway and a tolerance-associated immune checkpoint (21, 54).

AhR signaling provides a mechanistic link among coronavirus infection, Trp-derived ligands, and host-directed antiviral intervention. Experimental evidence indicates that AhR signaling is induced during infection with multiple coronaviruses and that pharmacological inhibition of AhR can reduce replication of human coronavirus 229E (HCoV-229E) and SARS-CoV-2 *in vitro*. Independent work extended this evidence across SARS-CoV-2 variants: AHR knockout or pharmacological antagonism reduced viral replication *in vitro*, and AhR antagonists lowered viral burden and lung inflammation in infected hamsters. These findings strengthen the argument that the Kyn–AhR axis may function not only as a severity-associated biomarker but also as a potentially exploitable host pathway in selected coronavirus settings (55–57).

Persistent alterations in Trp metabolism may also be relevant to post-acute COVID-19. Because the Kyn pathway intersects with serotonin availability, nicotinamide adenine dinucleotide (NAD) metabolism, and neuroactive metabolites such as quinolinic acid and kynurenic acid, as well as chronic immune activation, sustained pathway dysregulation could contribute to fatigue, cognitive dysfunction, and persistent inflammatory symptoms. However, this interpretation remains hypothesis-generating, and longitudinal studies are needed to determine whether altered Trp metabolism is a causal driver of post-acute sequelae or a biomarker of unresolved inflammation (55–57).

DENV provides a complementary +ssRNA model in which activation of the Kyn pathway appears temporally linked to acute inflammation and recovery. In hospitalized DENV patients, neopterin levels and Kyn/Trp ratios were significantly increased during acute disease and then decreased after recovery, supporting IFN-γ-associated immune activation and reversible IDO1 activity. This temporal pattern suggests that Kyn pathway activation is not inherently pathogenic; its biological significance depends on whether the response resolves as inflammation subsides or persists beyond the period of viral control (58–60).

DENV also illustrates why the pathway should not be reduced to a single Kyn/Trp ratio. Kyn, kynurenic acid, 3-hydroxykynurenine, quinolinic acid, and NAD precursors may have distinct effects on oxidative stress, endothelial activation, immune cell differentiation, and neuroinflammation. This is particularly relevant in dengue, where vascular leakage, platelet dynamics, cytokine tone, and endothelial dysfunction determine clinical severity. Future DENV studies should therefore combine viral load, cytokines, endothelial markers, and metabolite-specific Kyn profiling (58–60).

ZIKV is especially relevant because it links flavivirus replication, neurotropism, placental biology, and AhR-dependent host susceptibility. Giovannoni and colleagues showed that ZIKV infection induces Kyn production, activates AhR, and limits type I interferon responses. AhR inhibition suppresses ZIKV replication, and the same study also supports a proviral role for AhR in related DENV models. These data provide strong mechanistic support for positioning AhR as a host-directed antiviral target in selected flavivirus infections (27, 34).

The ZIKV model also shows that individual Kyn-pathway branches can support distinct antiviral mechanisms. Kyn-driven AhR activation may suppress antiviral interferon responses and support viral replication, whereas kynurenine-3-monooxygenase (KMO) and its enzymatic product, quinolinic acid, have been reported to trigger CaMKII/IRF3-mediated interferon-β production and broadly inhibit selected viral infections, including ZIKV, herpes simplex virus 1 (HSV-1), and SARS-CoV-2 models. Thus, the pathway should be analyzed as a branched immunometabolic network rather than a linear immunosuppressive cascade (27, 61).

Classical swine fever virus (CSFV) adds direct functional evidence at the IDO1 node. In infected PK-15 cells, IDO1 induction increased Trp catabolism, whereas IDO1 silencing or exogenous Trp enhanced NF-κB-dependent IFN-α, IFN-β, and IL-6 responses and reduced viral replication, including effects linked to autophagy. This places IDO1 as a functionally validated proviral regulator in a pestivirus model and supports *in vivo* confirmation (62).

HCV extends the +ssRNA model to chronic infection within a tolerogenic metabolic organ. In chronic HCV infection, hepatic IDO expression is upregulated, and serum Kyn/Trp ratios increase, supporting activation of Trp catabolism in both the liver and the periphery. Additional work on monocytes and dendritic cell populations from HCV patients indicates that IDO1/IDO2 expression is altered in chronic infection, linking persistent antigenic stimulation to immune-regulatory Trp metabolism (63, 64).

In HCV, the Kyn pathway should be interpreted through the lens of liver-specific immune tolerance. Persistent IDO1 activity may help limit immunopathology in inflamed hepatic tissue, but it may also contribute to ineffective antiviral T-cell responses and chronic low-grade inflammation. Because direct-acting antivirals have transformed HCV therapy, this virus is best used here as a chronic +ssRNA model of immune regulation and persistence rather than as a current therapeutic target for broad IDO1 blockade (42, 63, 64).

Chikungunya virus (CHIKV), an alphavirus, is an important emerging comparator and a priority for pathway-focused investigation. It is relevant to RNA-virus preparedness because CHIKV can cause acute infection followed by persistent inflammatory sequelae, but the current corpus contains limited direct evidence linking CHIKV to IDO1, Kyn metabolites, or AhR. Accordingly, CHIKV defines a focused opportunity for longitudinal immunometabolic profiling and experimental perturbation of IDO1, KMO, and AhR (15, 32, 42).

### 3.2 Negative-sense single-stranded RNA viruses: *in vivo* and cell-based evidence

IAV provides genetic, pharmacological, metabolic, and clinical evidence that the Kyn pathway shapes respiratory viral disease. IAV infection induces interferon-dependent IDO1 expression and stimulates Kyn biosynthesis, positioning Trp catabolism as part of the antiviral-inflammatory response. In contrast to settings where IDO1 is framed primarily as immunosuppressive, studies suggest that activation of the Kyn pathway may also serve as a negative feedback mechanism that restrains excessive pulmonary inflammation (19, 24).

In mice infected with PR8 or X31, Ido1 deletion reduced morbidity, accelerated recovery, and strengthened virus-specific CD8+ T-cell responses; D-1-methyltryptophan also accelerated recovery, although primary viral-clearance kinetics were unchanged. More recently, IDO1 knockdown reduced IAV-induced epithelial ferroptosis *in vitro*, while 1-methyltryptophan reduced acute lung injury and improved survival in infected mice. These studies establish IDO1 as a determinant of immune quality and disease severity in IAV and show why viral burden, immune function, and tissue injury should be measured together (65, 66).

Clinical and translational data support this phase-dependent interpretation. In patients hospitalized with seasonal influenza, neopterin and IDO activity have been evaluated as markers of immune activation and disease severity, whereas experimental models indicate that pharmacological manipulation of IDO during primary influenza infection can modify aspects of memory T-cell responses. Together, these observations make IAV a key model for the time-phase analysis because IDO1 can determine morbidity, recovery, antiviral T-cell quality, and pulmonary injury even when its effect on primary viral clearance is limited (65, 67, 68).

Influenza also illustrates that downstream Kyn metabolites may influence both pulmonary and neuroimmune outcomes. Reduced Trp availability may affect serotonin-related pathways, while accumulation of quinolinic acid and other metabolites may modulate inflammatory tone, oxidative stress, and tissue repair. Therefore, IAV should be interpreted not only as a respiratory infection model but also as a useful system for evaluating how acute viral inflammation reshapes systemic and neuroimmune metabolism (19, 69).

RSV provides direct cell-based evidence. RSV induces IDO expression and bioactivity in human monocyte-derived dendritic cells in a replication-dependent manner, suggesting that this virus can directly trigger Trp-catabolic immunoregulation. In airway epithelial cells, IDO has also been proposed to play a protective role during RSV-associated immune responses, supporting the idea that this pathway can limit inflammatory injury in the respiratory mucosa (6, 70, 71).

At the same time, RSV highlights unresolved complexity. In plasmacytoid dendritic cell models, RSV infection reduced kynurenic acid production and altered the Th17/Treg balance by modulating IDO, suggesting that pathway effects may vary by cell type, developmental context, and disease phase. RSV should therefore be presented as an emerging respiratory model in which IDO/Kyn biology may influence acute inflammation, immune memory, recurrent infection, and post-infectious airway remodeling, but longitudinal human metabolomics remains limited (15, 70–72).

### 3.3 Double-stranded RNA viruses: priority knowledge gaps and experimental opportunities

Rotavirus (RV), a segmented dsRNA virus, serves as a direct model for how intestinal epithelial cells detect viral RNA and mount protective interferon responses. In RV-infected intestinal epithelium, RIG-I/MDA5/MAVS signaling is required for these protective interferon responses, and purified RV RNAs can activate innate immune signaling via RIG-I-like receptors (73–75).

Mammalian orthoreovirus/reovirus (MRV/REOV) can serve as a second dsRNA comparator because reovirus RNAs are recognized by innate immune sensors, including the RIG-I and MDA5 pathways, and reovirus infection has been used to elucidate how host cells distinguish viral dsRNA from self-RNA. The strongest direct data for MRV/REOV currently establish innate RNA sensing and interferon biology; direct IDO1/Kyn/AhR perturbation is therefore the next decisive experiment. This makes MRV/REOV a valuable platform for testing dsRNA-induced immunometabolic rewiring (76, 77).

The conceptual value of dsRNA viruses lies in their ability to directly link viral RNA recognition to host-directed immunometabolism. If dsRNA sensing induces type I/III interferons and inflammatory cytokines, and if these signals are sufficient to trigger IDO1, GCN2-dependent nutrient stress, and Kyn-AhR signaling, then dsRNA viruses become experimentally useful systems for testing whether the Trp–Kyn axis is a conserved downstream module of RNA-virus sensing. This defines a testable hypothesis and a focused opportunity to extend pathway analysis to dsRNA infection (73, 74, 78).

### 3.4 Reverse-transcribing RNA viruses: causal murine and clinical evidence

LP-BM5 murine retrovirus provides a second causal *in vivo* model. Ido1 deletion or 1-methyl-D,L-tryptophan reduced proviral copies, increased type I interferon, and improved survival; neutralization of type I interferon restored viral replication. At the cellular level, IFN-γ-induced IDO1 has also been shown to restrict retrovirus infection through Trp depletion and autophagy. These complementary experiments identify IDO1 as an active determinant of retroviral infection at both systemic and cell-intrinsic levels (79).

HIV-1 remains the clearest model of sustained activation of the Kyn pathway during chronic viral infection. Early neuroimmunology studies showed that HIV-1-infected patients had reduced L-Trp levels, with proportional increases in L-Kyn and quinolinic acid in serum and cerebrospinal fluid. These metabolites correlated with immune activation markers, including neopterin and beta-2-microglobulin. This provides historical clinical evidence that chronic viral immune activation can drive compartmentalized Kyn metabolism (80–83).

Mechanistically, HIV-1-associated IDO1 activity contributes to an immune state in which inflammation and immunosuppression coexist. Favre and colleagues showed that IDO1-mediated Trp catabolism alters the balance between T helper 17 cells and regulatory T cells in HIV disease, linking microbial translocation, chronic immune activation, mucosal dysfunction, and regulatory skewing. This makes HIV-1 a key disease model for the maladaptive late phase of the Kyn switch, in which inflammatory tone remains high but effector antiviral immunity becomes functionally ineffective (84, 85).

Simian immunodeficiency virus (SIV) provides a comparative RT-RNA model alongside HIV-1, particularly when discussing tissue-specific Trp depletion and Kyn pathway activation across lymphoid, mucosal, and central nervous system compartments. Nevertheless, HIV-1 should remain the primary human disease anchor because it has stronger clinical evidence linking IDO1 activity, Kyn/Trp imbalance, Th17/Treg disruption, residual inflammation, neurocognitive effects, and inflammaging despite antiretroviral therapy (84–87).

### 3.5 Integrative interpretation across RNA virus classes

Taken together, the available evidence establishes the Kyn pathway as an active determinant of RNA-virus infection across multiple biological scales. Causal *in vivo* evidence is strongest in EMCV and LP-BM5, while IAV models demonstrate direct effects on morbidity, recovery, antiviral T-cell responses, and lung injury. Functional perturbation studies support roles for AhR in ZIKV, DENV, and coronaviruses; for IDO1 in CSFV and RSV; and for KMO/quinolinic acid across several RNA-virus cell systems. Human studies in COVID-19, dengue, influenza, HCV, and HIV further show that pathway activity tracks clinically relevant inflammation, immune dysfunction, or disease course (**Table 1**) (63, 73, 85).

**Table 1:** RNA virus classes, evidence strength, and Kyn-pathway involvement.

RNA virus class (Baltimore)	Virus	Evidence level, model, and pathway findings	Dominant interpretation	Host-directed implication	References
+ssRNA/IV	EMCV	Causal *in vivo*: Ido1 deficiency and 1-methyltryptophan increased survival, reduced viral burden, and increased type I interferon; metabolite rescue reversed protection. Kmo deficiency also modified mortality.	Proof of principle that IDO1/Kyn-pathway branches determine antiviral immunity and outcome.	Reference model for genetic–pharmacological–rescue studies and pathway-node selection.	(22, 23, 79).
	SARS-CoV-2	Clinical plus functional *in vitro*/*in vivo*: Reduced Trp, increased Kyn, elevated Kyn/Trp ratio; AhR signaling induced in coronavirus infection. AHR loss or antagonism reduces SARS-CoV-2 variant replication *in vitro* and viral burden and lung inflammation in hamsters.	Inflammatory burden plus possible AhR-mediated immune dysregulation.	Biomarker-guided IDO1/AhR modulation; AhR antagonism remains experimental.	(25, 28, 53).
	DENV	Longitudinal clinical plus functional *in vitro*: Increased neopterin and Kyn/Trp ratio during acute disease with reduction after recovery; AhR inhibition suppresses DENV in flavivirus models.	Phase-sensitive inflammatory marker; possible proviral AhR component.	Longitudinal metabolite profiling; experimental AhR blockade.	(26, 27).
	ZIKV	Functional *in vitro* and *in vivo*: ZIKV induces Kyn production and AhR activation; AhR inhibition suppresses infection; KMO/QUIN can enhance IFN-β antiviral signaling in selected models.	Tissue-specific complexity: AhR may be proviral, and downstream metabolites may be antiviral or neuroactive.	AhR inhibition and KMO modulation require timing and pregnancy- and tissue-aware design.	(28, 78).
	CSFV	Functional *in vitro*: IDO1 induction promoted Trp catabolism; IDO1 silencing or exogenous Trp increased NF-κB-dependent cytokine responses and reduced viral replication in PK-15 cells.	Direct proviral role for IDO1 linked to interferon regulation and autophagy.	Validate *in vivo* and define whether the mechanism extends to other pestiviruses or members of Flaviviridae.	(62).
	HCV	Clinical and ex vivo: Upregulated hepatic IDO expression; increased serum Kyn/Trp ratio; altered IDO expression in monocytes and dendritic-cell populations.	Chronic liver immune regulation and possible persistence-supporting tolerance.	Use as chronic +ssRNA model; therapeutic claims cautious in DAA era.	(63, 64).
	CHIKV	Priority evidence gap: Limited direct IDO1/Kyn/AhR evidence in the current corpus.	Knowledge gap relevant to chronic inflammatory sequelae.	Future immunometabolic profiling target.	(15, 42).
-ssRNA/V	IAV	Genetic/pharmacological *in vivo* plus clinical: IAV induces interferon-dependent IDO1 and Kyn biosynthesis; neopterin/IDO activity studied in hospitalized influenza patients.	Causal influence on morbidity, recovery, antiviral CD8+ T-cell responses, and lung injury; primary viral clearance is not the only relevant endpoint.	Use as an *in vivo* model to align IDO1 modulation with viral, immune, and tissue-injury endpoints.	(19, 24).
	RSV	Functional cell-based: RSV induces IDO expression and activity; IDO may protect airway epithelium; KYNA and Th17/Treg balance are altered in pDC models.	Emerging respiratory IDO/Kyn model; acute protection versus altered immune memory remains unresolved.	Longitudinal airway studies; phase-specific IDO/AhR modulation.	(70, 71).
dsRNA/III	Rotavirus	Indirect mechanistic evidence: RV RNA activates RIG-I/MDA5/MAVS and type I IFN signaling; direct KP evidence remains limited.	Upstream dsRNA-sensing model for IFN-driven immunometabolism.	Ideal platform to test dsRNA → IFN → IDO1/GCN2/AhR experimentally.	(73, 74).
	MRV/REOV	Indirect mechanistic evidence: Innate immune sensors recognize reovirus RNAs; direct evidence for KP remains limited.	Mechanistic comparator for dsRNA-triggered host-directed antiviral pathways.	Future model to test conserved RNA-sensing-to-Kyn coupling.	(76, 77).
RT-RNA/VI	LP-BM5	Causal *in vivo*: Ido1 deletion or 1-methyltryptophan reduced proviral copies, increased type I interferon, and improved survival; IFN-I neutralization restored replication.	Direct IDO1–IFN-I control of retroviral replication and disease.	Mechanistic bridge between murine causal evidence and human HIV/SIV studies.	(79).
	HIV-1	Clinical and ex vivo: Reduced Trp, increased Kyn/QUIN/KYNA in serum/CSF; IDO1 activity alters Th17/Treg balance; persistent pathway activation in chronic infection.	Chronic maladaptive activation: inflammation and immunosuppression coexist.	Model for residual inflammation, exhaustion, neuroimmune dysfunction, and late-phase maladaptive KP.	(84, 86, 88, 89).
	SIV	Comparative animal evidence: Comparative lentiviral model for compartmentalized Trp metabolism and chronic immune activation.	Tissue-specific chronic immunometabolic dysfunction.	Preclinical model for tissue- and phase-specific interventions.	(85–87).

The evidence is therefore most developed for selected +ssRNA, -ssRNA, and retroviral models, whereas CHIKV and dsRNA viruses remain priority areas for direct pathway testing. This gap defines the next experimental agenda: studies should identify the operative enzyme and metabolite branch; distinguish infected, bystander, stromal, and immune-cell compartments; resolve tissue and temporal dynamics; and measure viral burden together with interferon signaling, immune-cell function, and tissue injury. Concordant genetic, pharmacological, and metabolic-rescue approaches, complemented by longitudinal clinical metabolomics, will determine whether the pathway can be exploited in additional viral infections (21, 27, 28, 32).

## 4 Therapeutic window and the time-phase model

**Figure 4:**
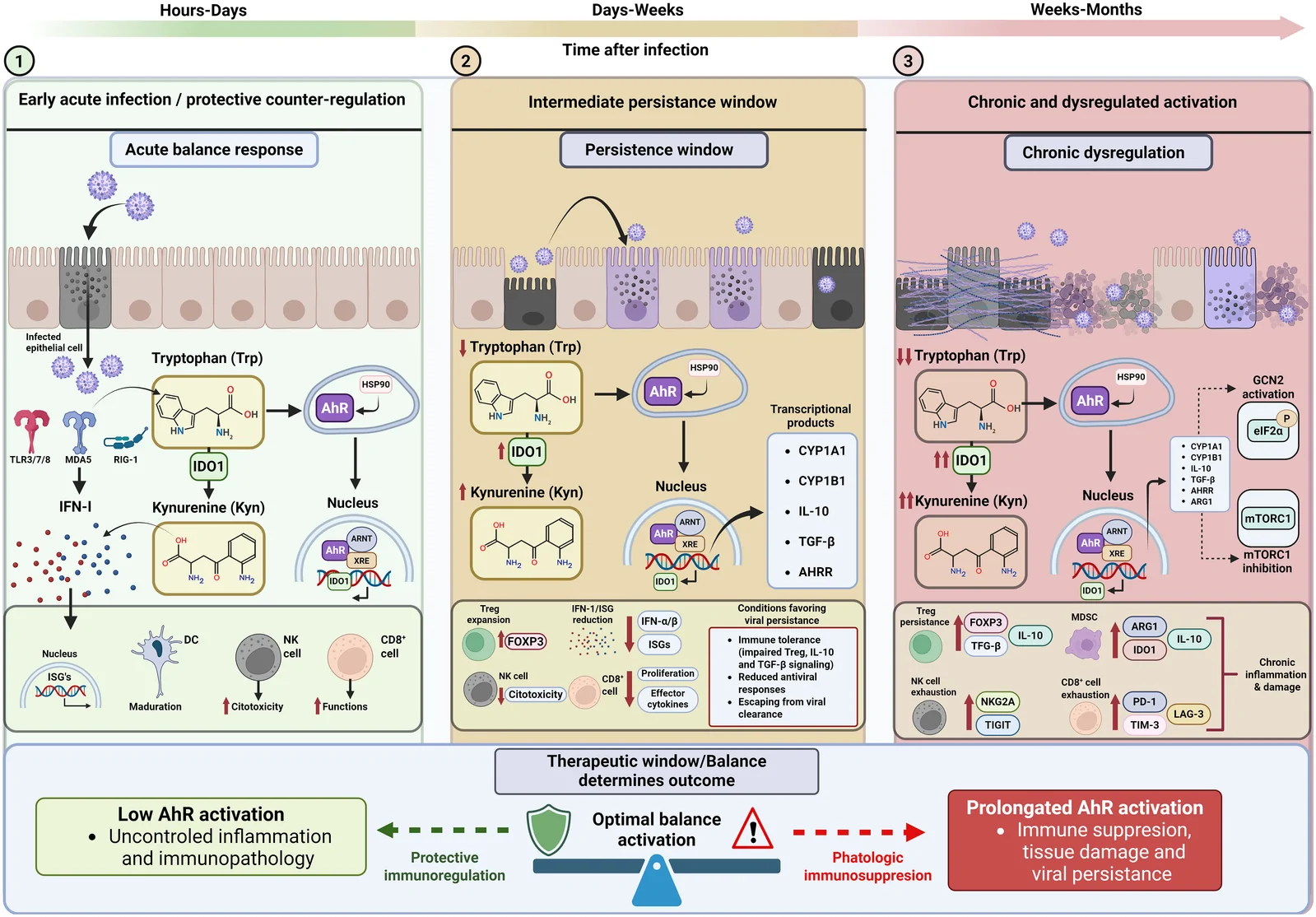
Time-phase model of Trp–Kyn–AhR regulation during RNA virus infection. The proposed model depicts the context- and time-dependent transition of the Trp–Kyn–AhR axis across three major stages of RNA virus infection. (1) Early acute infection: inflammatory and interferon signaling induces Trp catabolism, while limited and appropriately regulated pathway activation may restrain excessive inflammation without compromising viral clearance. (2) Intermediate persistence window: sustained IDO1 activity, progressive Trp depletion, Kyn accumulation, and AhR signaling may increasingly favor regulatory immune programs, reduced antiviral effector function, and conditions permissive to viral persistence. (3) Chronic or dysregulated activation: prolonged IDO1–Kyn–AhR signaling, together with GCN2 activation and mTORC1 inhibition, may contribute to immune dysfunction, chronic inflammation, tissue damage, and persistent or post-acute disease. The biological outcome is determined by the balance between insufficient immunoregulation, which may promote immunopathology, and excessive or prolonged pathway activation, which may impair antiviral immunity. This balance defines a phase-dependent therapeutic window for targeting the pathway.

The evidence summarized above shows that the Trp-Kyn-AhR axis can alter virological, immunological, and disease outcomes; translating that biological importance requires defining when and where each pathway node is active. The same pathway that supports inflammatory control and tissue protection in one phase of infection may impair antiviral immunity in another (15, 42). To reconcile these observations, we propose a time-phase model in which the functional impact of the Trp–Kyn–AhR axis depends on the stage of infection, viral burden, tissue damage, and the dominant immune program (**Figure 4**) (21, 32).

In this model, the pathway acts as an immunometabolic switch rather than a unidirectional suppressive circuit. During early infection, when viral replication and infected-cell clearance are the primary priorities, excessive IDO1 activity, Trp depletion, GCN2 activation, and Kyn-AhR signaling may dampen the antiviral response. In contrast, during later inflammatory or resolution phases, the same pathway may be beneficial by limiting cytokine-driven damage, supporting regulatory immune circuits, and promoting epithelial or tissue repair (27, 41).

### 4.1 Early infection: when immune restraint may favor viral persistence

In the early phase of RNA virus infection, the host must rapidly detect viral replication, produce type I and type III interferons, activate NK cells, prime antigen-presenting cells, and expand virus-specific T-cell responses. These events are essential for limiting viral spread before infection becomes systemic or establishes tissue reservoirs. Premature or excessive activation of the Trp-Kyn-AhR axis may be detrimental if it suppresses immune programs required for viral clearance (27, 28).

IDO1 induction during early infection may reduce local Trp availability and create a metabolically restrictive microenvironment. Although Trp depletion may theoretically limit protein synthesis and contribute to antimicrobial restriction, its effects on highly proliferative immune cells are particularly important because activated T cells require adequate aminoacid availability to sustain clonal expansion, cytokine production, and cytotoxic differentiation (41, 48, 49).

In parallel, Kyn accumulation may activate AhR-dependent transcriptional programs that favor regulatory or tolerogenic immune states. In early infection, this may reduce the intensity of NK-cell activity, CD8+ T-cell expansion, antigen-presenting-cell activation, and type I interferon-associated antiviral programs. Under these conditions, the pathway may create a window of reduced immune pressure that allows infected cells to persist longer, viral replication to continue, and adaptive immunity to develop less efficiently (27, 28, 31).

This early-phase risk does not imply that IDO1 or AhR activation is universally harmful at the onset of infection. Rather, it suggests that the intensity and timing of activation are decisive. A transient level of metabolic regulation may prevent excessive bystander inflammation, but dominant or premature tolerogenic signaling may reduce antiviral competence before viral control is achieved (15, 21, 32).

### 4.2 Acute inflammatory amplification: when the pathway reflects severity

As infection progresses, a high viral burden and tissue damage can amplify inflammatory signaling. Pattern-recognition receptors, inflammasome activation, interferons, IL-1, IL-6, TNF, and IFN-γ may converge to induce IDO1 and increase Kyn pathway activity. At this stage, elevated Kyn/Trp ratios may reflect the intensity of systemic inflammation rather than a clearly protective or pathogenic mechanism (19).

This is particularly relevant in severe acute infections such as COVID-19 and dengue, where altered Trp metabolism has been linked to inflammatory severity, lymphocyte dysfunction, vascular or pulmonary complications, and poor clinical outcomes. In these settings, the pathway may be viewed as part of a compensatory response to excessive inflammation, but such compensation does not necessarily confer protection if viral clearance remains incomplete (25, 90, 91).

This phase is the most challenging to interpret therapeutically. Blocking IDO1 or AhR too early might enhance antiviral effector activity, but doing so during an intense inflammatory wave could remove a regulatory brake, worsening tissue damage. Conversely, enhancing AhR-mediated tolerance during acute inflammation may reduce cytokine-driven injury, but it could impair residual antiviral clearance if viral replication persists (25, 90, 91).

### 4.3 Late infection and resolution: when immune regulation becomes protective

During the late phase of infection, viral burden may decline while inflammatory damage, epithelial barrier disruption, fibrosis, endothelial activation, or neuroimmune dysfunction may persist. In this context, the Trp-Kyn-AhR axis may shift from a potential suppressor of antiviral immunity to a homeostatic mechanism that limits immunopathology and promotes tissue repair (92–95).

AhR signaling is particularly relevant at this stage because it regulates barrier integrity, IL-22 production, antioxidant responses, epithelial differentiation, and tissue-remodeling programs. In respiratory and intestinal tissues, controlled AhR activation may support epithelial repair and reduce excessive inflammatory permeability. In the lung, AhR-dependent effects on fibroblast activation, TGF-β signaling, and matrix remodeling may influence the balance between repair and fibrosis (31, 51, 96).

IAV infection illustrates the protective aspect of the pathway. Although increased Kyn/Trp ratios may correlate with disease severity, IDO1 and Kyn pathway activity may also help limit late pulmonary immunopathology. This suggests that inhibiting the pathway during late inflammatory lung disease may be harmful if it functions as a negative feedback mechanism that restrains tissue injury (19, 24, 32, 97).

The late phase, therefore, highlights the therapeutic risk of oversimplification. A pathway that appears immunosuppressive during early viral replication may become tissue-protective during resolution. In this context, therapeutic approaches should aim to preserve or restore homeostatic regulation rather than indiscriminately suppressing IDO1 or AhR activity (31).

### 4.4 Chronic or persistent activation: when regulation becomes maladaptive

If activation of the Trp-Kyn-AhR axis persists, the pathway may become maladaptive. Chronic or sustained activation can create a state in which immune regulation, low-grade inflammation, and incomplete antiviral control coexist. This pattern is particularly evident in chronic infections such as HIV and HCV and may also be relevant to post-acute viral syndromes (42, 84).

In chronic infection, persistent IDO1 activity sustains Trp depletion and increases Kyn production. This can restrict T-cell proliferation, favor regulatory immune programs, impair cytotoxic responses, and reinforce exhaustion-like states. At the same time, sustained AhR activation may alter chemokine expression, reduce effector-cell recruitment, and promote tolerogenic myeloid or regulatory T-cell responses (62, 98).

HIV infection is a strong example of this pattern. Elevated IDO1 activity and increased Kyn/Trp ratios have been linked to chronic immune activation, T-cell dysfunction, frailty, and inflammaging. In this context, the pathway may no longer function as a transient protective brake but instead as part of a chronic immunometabolic loop that contributes to immune exhaustion, systemic inflammation, and impaired immune restoration (99, 100).

Post-acute viral syndromes may represent another form of maladaptive persistence. In conditions such as post-acute COVID-19, sustained alterations in Trp metabolism may contribute to fatigue, neurocognitive symptoms, immune dysregulation, or altered NAD metabolism, although causality remains uncertain. Future longitudinal studies are needed to determine whether persistent pathway activation drives symptoms, serves as a marker of unresolved inflammation, or is a compensatory response to tissue damage (45, 101, 102).

### 4.5 Therapeutic implications of the time-phase model

The time-phase model has clear but conditional therapeutic implications. It suggests that interventions targeting the Trp-Kyn-AhR axis should not be framed as a simple binary choice between inhibiting and activating the pathway. Instead, the key therapeutic questions are when, where, and in whom the pathway should be modulated (33).

During early infection, when viral replication is high and antiviral effector activity is insufficient, transient inhibition of IDO1 or AhR may theoretically restore immune pressure by improving NK-cell function, preserving CD8+ T-cell expansion, and preventing premature tolerogenic programming. However, during acute hyperinflammatory disease, pathway inhibition may be risky unless viral replication remains the dominant driver of pathology (92, 103).

During the late inflammatory or tissue-repair phases, controlled AhR activation or preservation of IDO1-dependent regulatory circuits may help limit immunopathology and promote repair. In chronic or post-acute settings, therapeutic decisions may hinge on whether the pathway is sustaining immune dysfunction, supporting tissue recovery, or merely reflecting unresolved inflammation (29–32, 42, 84).

This framework also underscores the need for biomarkers. The Kyn/Trp ratio may offer a useful but incomplete estimate of pathway activity. More informative approaches should integrate downstream metabolite profiling, IDO1/IDO2/TDO expression, AhR transcriptional signatures, viral load kinetics, interferon-stimulated gene expression, immune-cell phenotyping, and tissue-specific markers of injury or repair (32).

Ultimately, the time-phase model reframes the Kyn pathway as a dynamic regulator of host-virus interactions. Its biological output depends on the temporal relationships among viral replication, immune activation, metabolic stress, and tissue damage. This model provides the conceptual basis for the pharmacological strategies discussed in the next section, in which IDO1 inhibitors, AhR agonists and antagonists, and Trp-based antiviral approaches are considered tools that must be aligned with disease phase and immunological context (15, 32, 42).

## 5 Pharmacological targeting of the Trp-Kyn-AhR axis

The time-phase model proposed above offers a therapeutic framework in which the Trp-Kyn-AhR axis is viewed as a dynamic immunometabolic circuit rather than a target to be globally inhibited or activated. Pharmacological intervention should therefore account for disease phase, viral burden, inflammatory intensity, tissue compartment, and dominant immune phenotype (32, 33). Within this framework, IDO1 inhibitors, AhR modulators, KMO-directed strategies, and Trp-based entry inhibitors represent distinct therapeutic approaches with different biological objectives (31, 51, 96, 104, 105).

### 5.1 IDO1 inhibitors: restoring antiviral competence

IDO1 is the most extensively studied pharmacological node of the Kyn pathway. By catalyzing Trp degradation and promoting Kyn accumulation, IDO1 can link inflammation to amino acid deprivation, GCN2 activation, AhR signaling, mTORC1 restraint, and reduced effector immune activity (32, 46). These mechanisms have supported the development of IDO1 inhibitors, including epacadostat, indoximod, navoximod, and linrodostat, which differ in selectivity, reversibility, catalytic mechanism, and tissue distribution (33).

However, the inconsistent performance of IDO1 inhibitors in cancer immunotherapy suggests that blocking IDO1 enzymatic activity does not necessarily confer clinical benefit. Compensatory IDO2 or TDO activity, non-catalytic IDO1 functions, inadequate patient selection, and poor alignment between pathway activity and therapeutic timing may all limit efficacy (32, 46). These lessons are directly relevant to viral infections, where IDO1 activity may reflect either maladaptive immunosuppression or protective inflammatory restraint.

In early infection or chronic immune dysfunction, excessive IDO1 activity may compromise antiviral pressure by limiting Trp availability, weakening mTORC1-dependent lymphocyte expansion, and reducing CD8+ T-cell and NK-cell function. In such settings, transient IDO1 inhibition could theoretically enhance cytotoxic activity and viral clearance. By contrast, in late inflammatory or resolution phases, IDO1 may limit tissue-damaging inflammation; its blockade could therefore amplify immunopathology rather than improve antiviral control (33). Thus, IDO1 inhibitors should be considered phase-specific immunometabolic tools rather than broad antiviral or anti-inflammatory agents.

### 5.2 AhR modulation: antagonism or agonism according to biological need

AhR is the primary transcriptional effector through which Kyn and other Trp-derived metabolites regulate immune and tissue responses. Therapeutic modulation of AhR is complex because AhR activation may support epithelial barrier function, IL-22 production, antioxidant responses, tissue repair, antifibrotic programs, and immune tolerance, but may also impair antiviral IFN responses, cytotoxic activity, and viral clearance in selected contexts (29, 31, 51, 96).

During early infection, excessive Kyn-AhR signaling may be detrimental if it suppresses type I IFN responses, NK-cell activity, CD8+ T-cell function, or antigen-presenting-cell activation before viral control is achieved. In ZIKV and coronavirus models, AhR activation has been linked to impaired antiviral defense or enhanced viral replication, whereas AhR inhibition reduced viral burden in selected experimental systems (27, 28). AhR antagonists such as CH223191, BAY-2416964, IK-175, and clofazimine therefore serve as useful pharmacological tools to test whether AhR blockade can restore antiviral pressure in defined viral contexts (31, 96, 106, 107).

Conversely, AhR agonism may be beneficial when tissue repair or inflammatory resolution predominates. Dietary indoles, microbiota-derived ligands, FICZ, quercetin, indole-3-carbinol, 3,3-diindolylmethane, and tapinarof support the broader concept that controlled AhR activation can promote barrier restoration, epithelial differentiation, IL-22-dependent repair, antioxidant responses, and inflammatory control (29, 31, 51, 96, 106, 107). AhR may also influence post-inflammatory remodeling by interfering with TGF-β1 signaling, p-Smad2/3 nuclear translocation, fibroblast-to-myofibroblast differentiation, and extracellular matrix remodeling (30, 31, 96). Therefore, AhR should not be defined as intrinsically proviral or antiviral; its value depends on whether the therapeutic objective is immune activation, viral clearance, barrier repair, or inflammatory containment.

### 5.3 KMO modulation and downstream Kyn metabolite rewiring

The Kyn pathway should not be reduced to IDO1 activity or the Kyn/Trp ratio. Downstream metabolism yields kynurenic acid, 3-hydroxykynurenine, quinolinic acid, and NAD-related metabolites, each of which may influence oxidative stress, neuroinflammation, IFN signaling, mitochondrial function, and tissue homeostasis (32, 78). Pharmacological modulation of enzymes such as KMO may therefore redirect pathway output rather than suppress Trp degradation.

This is relevant because downstream metabolites may have divergent antiviral or pathogenic effects. Kyn-driven AhR activation may suppress antiviral IFN responses in selected ZIKV and coronavirus models. In contrast, KMO activity and quinolinic acid production have been linked to CaMKII/IRF3-mediated IFN-β induction and antiviral activity in experimental systems. In contrast, sustained accumulation of neuroactive metabolites, such as quinolinic acid, may contribute to excitotoxicity, oxidative stress, or neuroinflammation in neurotropic or post-acute viral syndromes (27, 28, 78). KMO-directed strategies should therefore be considered emerging, tissue-specific approaches that require metabolite-level validation in RNA virus infection.

### 5.4 Trp-based oligomers and viral entry inhibition

Trp-based entry inhibitors are conceptually distinct from IDO1-GCN2-AhR immunometabolic modulation. In this context, Trp is used as a chemical scaffold with aromatic, hydrophobic, and amphipathic properties that can interfere with viral attachment, receptor engagement, or membrane fusion (104, 108–110). This mechanism is most relevant during prophylaxis or early infection, before genome replication is established.

Several RNA virus models support this concept. Trp trimers and tetramers, including AL439 and AL440, inhibit DENV and ZIKV attachment at low micromolar concentrations (104). Interfacially active peptides can disrupt viral particles or interfere with membrane-dependent steps of DENV and ZIKV entry (108). In enterovirus A71, dendrimers such as MADAL385 block receptor engagement mediated by PSGL1 and heparan sulfate, while simplified Trp oligomers linked to pentaerythritol scaffolds show activity against enterovirus A71 and HIV-1 (111). In SARS-CoV-2, thiophenyl-substituted Trp trimers and tetramers reduce cellular entry *in vitro*, likely through interaction with Spike. However, the functional relevance of L-Trp interaction with the receptor-binding domain requires further validation (110, 112).

These compounds should be presented as part of an adjacent Trp-related antiviral strategy rather than as direct Kyn-pathway modulators. This distinction is important experimentally because Trp-related interventions may reduce viral replication either indirectly through host immunometabolism or directly by blocking viral entry.

### 5.5 Biomarker-guided and combination strategies

Because the Trp-Kyn-AhR axis can play protective, maladaptive, or compensatory roles, therapeutic decisions require biomarkers that define pathway state rather than pathway presence alone (32, 33). The Kyn/Trp ratio is a useful indirect estimate of pathway activation. Still, it cannot, by itself, distinguish antiviral inflammation from immune regulation, severe systemic inflammation, or chronic immune dysfunction (25, 33, 90).

A more informative strategy should integrate Trp, Kyn, kynurenic acid, 3-hydroxykynurenine, quinolinic acid, and NAD-related metabolites; IDO1, IDO2, and TDO expression; AhR transcriptional signatures; ISGs; viral load kinetics; lymphocyte counts; NK-cell and CD8+ T-cell functional markers; inflammatory cytokines; and tissue injury or repair markers (32, 33). Longitudinal sampling will be essential because single-time-point measurements cannot determine whether pathway activation is rising, resolving, or persisting abnormally (26, 42, 84, 90). Molecular imaging, including IDO1-directed positron emission tomography, may further help map tissue-specific Trp catabolism *in vivo*, although this approach remains investigational (33).

Future strategies may require combinations pairing immunometabolic modulation with direct antivirals, IFN modulators, immune checkpoint inhibitors, anti-inflammatory agents, or tissue-protective interventions (32, 33, 105). IDO1 inhibitors may be most rational when combined with direct-acting antivirals in settings of active replication and impaired effector immunity. AhR antagonists may be appropriate when AhR signaling suppresses IFN responses or T-cell function. In contrast, AhR agonists or barrier-protective ligands may be preferable during resolution phases marked by epithelial injury or tissue remodeling (27–29, 31).

**Figure 5:**
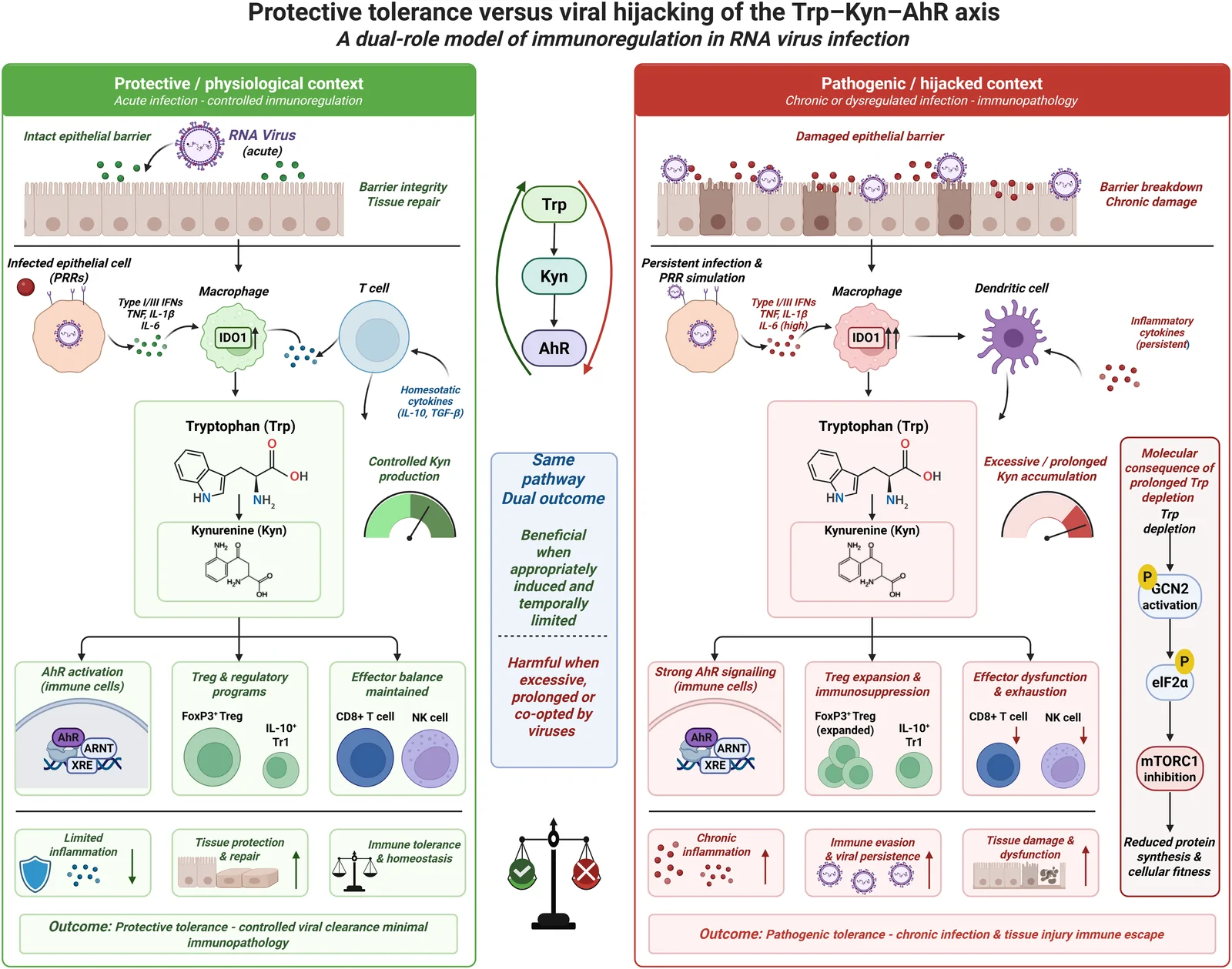
Protective tolerance versus viral hijacking of the Trp–Kyn–AhR axis. Dual conceptual model illustrating the context-dependent outcomes of Trp–Kyn–AhR signaling in RNA virus infection. Under physiological or acute conditions, transient pathway activation limits excessive inflammation, preserves epithelial barrier integrity, promotes tissue repair, and maintains immune homeostasis while enabling effective viral clearance. Conversely, persistent or excessive activation drives prolonged Trp depletion, sustained AhR signaling, immune-cell exhaustion, regulatory T-cell expansion, impaired antiviral effector responses, chronic inflammation, tissue injury, and viral persistence. The biological outcome depends primarily on the magnitude and duration of pathway activation.

These therapeutic possibilities are summarized in **Table 2**, which organizes the major pharmacological nodes of the Trp-Kyn-AhR axis by therapeutic window, mechanistic objective, timing-related risk, biomarker requirements, and supporting references. This framework emphasizes that the central therapeutic question is not merely whether to inhibit or activate the pathway, but when, where, and in whom to modulate it. The dual biological consequences of Trp–Kyn–AhR signaling are summarized conceptually in **Figure 5**.

**Table 2:** Phase-dependent pharmacological strategies targeting the Trp-Kyn-AhR axis in RNA virus infection.

Therapeutic node	Representative approaches	Most plausible window	Main rationale	Major risk if mistimed	Key biomarkers	References
IDO1 inhibition	Epacadostat, indoximod, navoximod, linrodostat	Early infection or chronic immune dysfunction	Limits Trp depletion and Kyn accumulation; may restore CD8+ T-cell, NK-cell, IFN-γ, and mTORC1-dependent responses	May worsen late immunopathology by removing a homeostatic brake	Kyn/Trp ratio, IDO1 expression, viral load, CD8+/NK function, IFN signatures	(33, 46, 105).
AhR antagonism	CH223191, BAY-2416964, IK-175, clofazimine	Early infection with proviral AhR activity	May restore type I IFN signaling and reduce tolerogenic or exhaustion-like programs	May impair barrier repair, IL-22 responses, and inflammatory resolution	AhR target genes, Kyn/AhR ligands, ISGs, viral load, T-cell dysfunction markers	(27, 28, 31, 96, 106, 107).
AhR agonism	FICZ, dietary indoles, microbiota-derived ligands, quercetin, indole-3-carbinol, 3,3-diindolylmethane, tapinarof	Late inflammation or resolution	Supports epithelial repair, barrier integrity, IL-22, antioxidant responses, and tissue protection	May suppress antiviral IFN and cytotoxic responses during active replication	Declining viral load, tissue-injury markers, epithelial barrier markers, and inflammatory cytokines	(29, 31, 51, 96, 106, 107).
KMO/downstream metabolite modulation	KMO-directed strategies; metabolite rewiring	Neurotropic, systemic, or post-acute contexts	Redirects kynurenic acid, quinolinic acid, 3-hydroxykynurenine, and NAD-related outputs	May shift metabolism toward neurotoxic or immunosuppressive metabolites	Full Kyn metabolomics, NAD metabolites, neuroinflammatory, and tissue-injury markers	(27, 28, 32, 78).
Trp-based entry inhibition	Trp trimers/tetramers, dendrimers, Trp-rich scaffolds	Prophylaxis or early entry phase	Directly interferes with viral attachment, receptor engagement, or membrane fusion.	Limited utility after replication is established; virus- and envelope-dependent activity.	Entry-stage assays, viral attachment/fusion readouts, and low-established viral burden	(104, 108–111, 113).
Biomarker-guided combinations	IDO1/AhR modulation plus antivirals, IFN modulators, anti-inflammatory or tissue-protective agents	Phase-defined precision therapy	Aligns immune restoration, viral suppression, and tissue protection	Poor timing may impair clearance or amplify immunopathology	Kyn/Trp, metabolomics, IDO1/IDO2/TDO, AhR signature, viral load, ISGs, immune phenotype	(32, 42, 84, 107).

## 6 Conclusions and future perspectives

The Trp–Kyn–AhR axis is an important immunometabolic regulator of RNA virus infection. Causal evidence from EMCV and LP-BM5, *in vivo* IAV studies, functional perturbation of IDO1, KMO, or AhR, and human association studies indicate that this pathway can influence viral burden, interferon responses, immune-cell function, tissue injury, and survival. Because these outcomes may diverge across models, biological relevance should not be inferred from a uniform phenotype across infections. Accordingly, increased IDO1 expression or elevated Kyn/Trp ratios should be interpreted as evidence of pathway engagement rather than as direct markers of antiviral activity or pathogenicity. Mechanistic interpretation requires integration with viral kinetics, interferon responses, immune-cell function, tissue-injury markers, and compartment-specific metabolite measurements (25, 26, 90).

Our proposed time-phase model provides a testable framework in which pathway function is evaluated across defined stages and tissues rather than inferred retrospectively from disease severity. It predicts that the same node may impair antiviral pressure before viral clearance yet promote disease tolerance, barrier integrity, or tissue repair when immunopathology predominates (27, 28).

Therapeutic translation therefore depends on when, where, and in whom the pathway is targeted. IDO1 inhibitors, KMO modulators, or AhR antagonists should be prioritized only when genetic and pharmacological evidence converge and when target engagement, PK/PD, virological efficacy, and tissue-level effects are demonstrated in relevant models.

Future studies should focus on underexplored RNA viruses and combine longitudinal metabolomics with viral load, interferon signaling, spatial or cell-resolved profiling, and genetic or pharmacological rescue experiments. Such approaches will help distinguish shared, therapeutically actionable dependencies from virus-, tissue-, or phase-specific effects (15, 31). Post-acute disease also warrants dedicated investigation, as persistent IDO2/AhR activity, altered NAD metabolism, and neuroactive Kyn metabolites may reflect unresolved inflammation, compensatory responses, or causal pathology that can only be distinguished through longitudinal perturbation studies.

Overall, the Trp–Kyn–AhR axis is biologically well supported but therapeutically immature. Its immediate value lies in converting current mechanistic gaps into testable hypotheses, using EMCV as causal proof of principle while preserving virus- and context-specific biology to guide the development of antiviral immunometabolic strategies.
